# Adaptive Fifth-Degree Cubature Information Filter for Multi-Sensor Bearings-Only Tracking

**DOI:** 10.3390/s18103241

**Published:** 2018-09-26

**Authors:** Haonan Jiang, Yuanli Cai

**Affiliations:** School of Electronic and Information Engineering, Xi’an Jiaotong University, Xi’an 710049, China; jianghaonan@stu.xjtu.edu.cn

**Keywords:** cubature information filter, multi-sensor bearings-only tracking, covariance matching, sensor selection

## Abstract

Standard Bayesian filtering algorithms only work well when the statistical properties of system noises are exactly known. However, this assumption is not always plausible in real target tracking applications. In this paper, we present a new estimation approach named adaptive fifth-degree cubature information filter (AFCIF) for multi-sensor bearings-only tracking (BOT) under the condition that the process noise follows zero-mean Gaussian distribution with unknown covariance. The novel algorithm is based on the fifth-degree cubature Kalman filter and it is constructed within the information filtering framework. With a sensor selection strategy developed using observability theory and a recursive process noise covariance estimation procedure derived using the covariance matching principle, the proposed filtering algorithm demonstrates better estimation accuracy and filtering stability. Simulation results validate the superiority of the AFCIF.

## 1. Introduction

Bearings-only tracking (BOT), which is also named target motion analysis (TMA), has been widely used in military applications, such as underwater tracking, aircraft surveillance and electronic warfare [1]. As an efficient passive means, BOT tracks the target of interest without emitting electromagnetic waves, which can prevent the tracking system from being detected and improve the fighting capabilities. The goal of BOT is to acquire kinematic parameters of the target using bearing measurements corrupted by noise [2].

For single-sensor BOT problem, the sensor must outmaneuver the target to guarantee the observability of the tracking system. Therefore, how the sensor moves becomes a crucial factor that influences the tracking accuracy and many researchers have devoted themselves to this research topic [3,4,5,6,7]. However, it is always difficult and sometimes unrealistic to design an optimal trajectory for the sensor. Under the premise that the sensor moves according to a given trajectory, there are also many focusing on the tracking algorithms. Over the past decades, the evolution of Bayesian filtering technology provides BOT with numerous choices, among which the most important and frequently used method is the extended Kalman filter (EKF) [8,9,10]. Although simple to implement, tracking algorithms that are based on the EKF linearize the nonlinear system model by utilizing Taylor-series expansion, which may lead to unstable and even divergent filtering results. In recent years, sigma-point based filters, including deterministic-point-based ones that are represented by cubature Kalman filter (CKF) [11,12,13] and random-point-based ones represented by particle filter (PF) [14], have attracted much attention. Instead of approximating the nonlinear functions, like EKF, this kind of filters adopt different numerical rules to approximate the probability density distributions of nonlinear systems. When filtering accuracy, numerical stability, and computational demands are all considered, the CKF seems to be a desirable choice for state estimation in BOT. This is because of the evident drawbacks existing in other sigma-point based filters, including the possible invalidation of the unscented Kalman filter (UKF) if the number of system states is more than three and the heavy computational burden of quadrature Kalman filter (QKF) and PF, etc. The conventional CKF adopts the third-degree cubature rule, which can guarantee at least second-order Taylor precision [15]. In order to further improve filtering precision and numerical stability, a new class of CKFs based on the high-degree cubature rules [16] is developed.

With the quick development of electronic technology, sensors become low-cost and tracking systems equipped with multiple sensors are becoming increasingly preferable for BOT. The bearing measurements received by sensors at different locations can be fused, thus the system observability and tracking performance can both be improved significantly. Nonetheless, unobservable cases may still occur in multi-sensor BOT. Therefore, the sensors that are used for tracking the target should be selected properly and saving sensor network resource should also be put into consideration at the same time. So we should try to achieve the optimal tracking efficacy with the fewest sensors. The information filter [17], which is algebraically equivalent to the classical Bayesian filter, is more suitable for multi-sensor data fusion. In the information filtering framework, the estimated parameters are the information-form states and matrix. Compared with conventional filtering algorithms, the information-form counterparts [18,19,20] have the advantages of easy initialization, easy decentralization, and low computational burden.

The standard Bayesian filtering algorithms are efficient, provided only that the statistic of process noise is exactly known. In real combat scenarios, however, this assumption is not usually reasonable. Thus, adaptive filtering algorithms are needed to solve this model mismatching problem. This type of filters can be mainly divided into innovation-based ones and the ones using multiple models [21]. Innovation-based filters [21,22,23,24] adopt covariance matching method or maximum likelihood estimation to modify the statistic of the process noise. Drawbacks, such as heavy computation burden, non-positive matrix, and offline estimation prevent these methods from being utilized in reality. Since being proposed, the interacting multiple model (IMM) method [25] has been extensively used for state estimation under unknown system parameters, but its performance depends on the selection of model sets.

In this paper, we propose an adaptive nonlinear estimation approach, called the adaptive fifth-degree cubature information filter (AFCIF), which shows better performance than the standard algorithms for multi-sensor BOT especially when the statistic of process noise is unknown. It is built on the basis of the newly introduced fifth-degree CKF [16] and adopts the structure of information filter. A prominent feature of this algorithm is the recursive process noise covariance estimation procedure, which is derived using covariance matching principle [22]. This can address the issue of state estimation with unknown process noise covariance in BOT. In addition, a sensor selection strategy is presented to enhance the system observability. Various kinds of criteria [26,27,28,29] have been proposed for sensor selection in multi-sensor target tracking, including Fisher information, estimation error covariance, and maximum entropy, etc. Our strategy is based on maximizing the inverse of the condition number [30] of the observability matrix, intending to achieve the best observable condition with the given sensor network. Combined with the sensor selection strategy and the recursive process noise covariance estimation procedure, the novel algorithm demonstrates better adaptivity, filtering accuracy, and stability.

The rest of the paper is organized as follows. Section 2 describes the problems that we will solve in multi-sensor BOT. The Bayesian filtering theory is reviewed in Section 3. Section 4 derives the sensor selection strategy and Section 5 introduces the AFCIF algorithm for multi-sensor BOT. The convergence of the proposed algorithm is analyzed in Section 6. Simulations for a certain multi-sensor BOT scenario and performance analysis are given in Section 7. Section 8 summarizes our main contributions.

## 2. Problem Formulation

**Figure 1:**
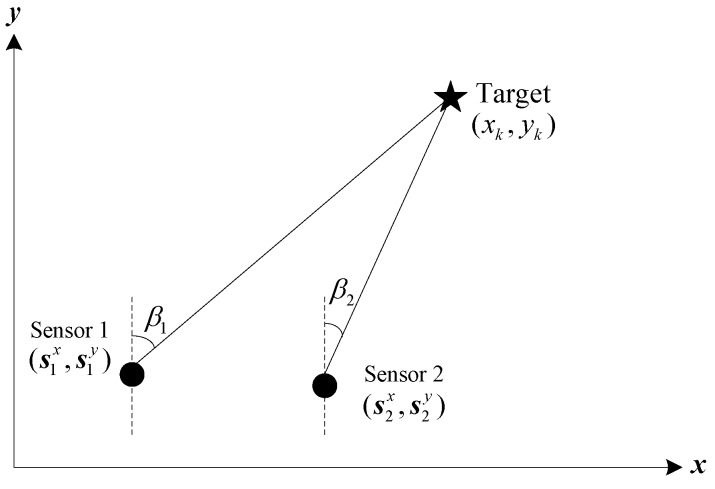
Geometric configuration of multi-sensor BOT.

The multi-sensor BOT problem in a two-dimensional (2-D) Cartesian coordinate plane is depicted in Figure 1. The position and velocity of the target at time *k* are denoted by $\left(x_{k},y_{k}\right)$ and $\left({\dot{x}}_{k},{\dot{y}}_{k}\right)$, and the state vector of the target at time *k* is defined as $x_{k}={\left[x_{k}\;y_{k}\;{\dot{x}}_{k}\;{\dot{y}}_{k}\right]}^{T}$, then the system model can be written as

(1) $$x_{k}=Fx_{k-1}+w_{k-1}\;$$

(2) $$z_{k}=h_{k}(x_{k})+v_{k}\;$$

where $x_{k}\in R^{n}$, n (=4) is the dimension of the state vector and F denotes the state transition matrix that decides the motion model of the target and is given by

(3) $$F=\left[\begin{array}{cccc} 1 & 0 & \Delta T & 0\\ 0 & 1 & 0 & \Delta T\\ 0 & 0 & 1 & 0\\ 0 & 0 & 0 & 1 \end{array}\right]\;$$

where ΔT represents the sample interval. $z_{k}\in R^{m}$ is the measurement vector at time *k* and m (=2) is its dimension. $h_{k}(\cdot )$ is the measurement function at time *k*, $w_{k-1}$ and $v_{k}$ represent process noise and measurement noise, respectively.

At time *k*, two sensors among the available sensor network are selected to measure the azimuths between the target and themselves. So $h_{k}(x_{k})$ can be written as follows:

(4) $$h_{k}(x_{k})=\left[\begin{array}{c} h_{k,1}(x_{k})\\ h_{k,2}(x_{k}) \end{array}\right]=\left[\begin{array}{c} \beta_{k,1}\\ \beta_{k,2} \end{array}\right]=\left[\begin{array}{c} \arctan(\frac{x_{k}-s_{1}^{x}}{y_{k}-s_{1}^{y}})\\ \arctan(\frac{x_{k}-s_{2}^{x}}{y_{k}-s_{2}^{y}}) \end{array}\right]\;$$

and

(5) $$v_{k}=\left[\begin{array}{c} v_{k,1}\\ v_{k,2} \end{array}\right]\;$$

where $\left(s_{i}^{x},s_{i}^{y}\right)$ and $v_{k,i}$ denote the location and measurement noise, respectively, of the *i*th sensor. $w_{k-1}$ and $v_{k,i}$ are assumed to be uncorrelated noises with Gaussian distributions $N(w_{k-1};0,Q_{k-1})$ and $N(v_{k,i};0,R_{i})$, and $v_{k}\sim N(0,R)$ with $R=\mathrm{diag}(R_{1},R_{2})$.

**Problem** **1.** *The process noise covariance is assumed to be time-invariant but unknown. In this case, standard filtering algorithms will collapse and some improvements should be made on them.*

**Problem** **2.** *At each time step k, given a set of sensors at different fixed locations,*$S=\{s_{1},s_{2},\cdots ,s_{N_{k}}\}$*,*$s_{i}=(s_{i}^{x},s_{i}^{y}),\;i=1,2,\cdots ,N_{k}$*and the state estimates of the target*$x_{k}$, the sensor selection problem can be formulated as

(6) $$\begin{array}{c} \underset{s_{1},s_{2}}{\max}OD(s_{1},s_{2})\\ \mathrm{subject}\;\mathrm{to}\;s_{i}\in S,\;i=1,2. \end{array}$$

*where*$OD(s_{1},s_{2})$*denotes the observability degree of the BOT system when*$s_{1},s_{2}$*are selected to track the target.*

**Remark** **1.** *In this paper, we only consider the problem of tracking the target using the bearing measurements from two selected sensors, which is a special case of multi-sensor BOT. With the number of passive sensors increasing, the system observability and tracking performance will be enhanced gradually. Therefore, the two-sensor case seems to be the most difficult in multi-sensor BOT and worth studying.*

## 3. Bayesian Filtering

The aim of BOT is to acquire estimates of the target state $x_{k}$ given a set of measurements $z_{1:k}$. Based on the posterior density $p(x_{k-1}|z_{1:k-1})$ at time k−1, the predicted density of the state can be firstly evaluated through one-step prediction in Bayesian filtering techniques

(7) $$p(x_{k}|z_{1:k-1})=\int p(x_{k}|x_{k-1})p(x_{k-1}|z_{1:k-1})dx_{k-1}\;$$

and then the posterior density at time *k* can be computed recursively by

(8) $$p(x_{k}|z_{1:k})=\frac{p(z_{k}|x_{k})p(x_{k}|z_{1:k-1})}{\int p(z_{k}|x_{k})p(x_{k}|z_{1:k-1})dx_{k}}\;$$

where $p(x_{k}|x_{k-1})$ is the state transition density and $p(z_{k}|x_{k})$ is the measurement likelihood.

It is obvious that the multidimensional integrals in Equations (7) and (8) are intractable if nonlinear relationships exist in the system model. Since the measurement function in the BOT system is nonlinear, the traditional Kalman filter (KF) could not be directly applied and nonlinear solutions are needed. By linearizing the nonlinear function in the system model, the EKF has the similar filtering procedure to KF. However, since the nonlinearity of the measurement function (arc tangent) in BOT system is severe, the performance of the EKF will be degraded significantly.

Instead of approximating the nonlinear function in the system model, the kind of deterministic-point-based filters approximate the probability density distribution of the system state through a group of deterministic weighted points. Under the assumption that the state is following the Gaussian distribution, the main goal of deterministic-point-based filters is to approximately calculate the multidimensional integrals, which can be expressed as the product of a nonlinear function and a Gaussian probability density, as follows:

(9) $$\begin{array}{cc} I(g) & =\int g(x)N(x;\hat{x},P)dx\\ & =\int g(\sqrt{P}x+\hat{x})N(x;0,I_{n})dx\\ & =\sum_{i=1}^{N_{\xi }}\omega_{i}g(\sqrt{P}\xi_{i}+\hat{x}) \end{array}$$

where g(⋅) is an arbitrary nonlinear function and $N(x;\hat{x},P)$ denotes a Gaussian distribution with mean $\hat{x}$ and covariance P. $\xi_{i}$ and $\omega_{i}$ are the chosen deterministic points and their corresponding weights, which can be generated by various rules, such as the unscented transform, the cubature rules, and the Gauss-Hermite quadrature rule. For more details of these rules, please refer to [15,16,18,19,20].

Taking both estimation precision and time complexity into account, we adopt the fifth-degree cubature rule [16] to approximate the multidimensional integrals. Since being proposed, this new rule for CKF has attracted a lot of attention in various research areas [31,32,33]. The set of $N_{\xi }=2n^{2}+1$ deterministically selected cubature points and their corresponding weights that are based on this rule are defined in Equations (10) and (11).

(10) $$\xi_{i}=\left\{ \begin{array}{ll} {\left(0,0,\cdots ,0\right)}^{T} & i=0\\ \;\sqrt{n+2}e_{i} & i=1,2,\cdots ,n\\ -\sqrt{n+2}e_{i-n} & i=n+1,\cdots ,2n\\ \;\sqrt{n+2}\eta_{i-2n}^{+} & i=2n+1,\cdots ,n(n+3)/2\\ -\sqrt{n+2}\eta_{i-n(n+3)/2}^{+} & i=n(n+3)/2+1,\cdots ,n(n+1)\\ \;\sqrt{n+2}\eta_{i-n(n+1)}^{-} & i=n(n+1)+1,\cdots ,n(3n+1)/2\\ -\sqrt{n+2}\eta_{i-n(3n+1)/2}^{-} & i=n(3n+1)/2+1,\cdots ,2n^{2} \end{array}\right.\;$$

(11) $$\omega_{i}=\left\{ \begin{array}{cc} \frac{2}{n+2} & i=0\\ \frac{4-n}{2{\left(n+2\right)}^{2}} & i=1,2,\cdots ,2n\\ \;\frac{1}{{\left(n+2\right)}^{2}} & i=2n+1,\cdots ,2n^{2} \end{array}\right.\;$$

where *n* is the dimension of the state vector, $e_{i}\in R^{n}$ is the unit vector with the *i*th component being 1, and

(12) $$\left\{\eta_{j}^{+}\right\}=\left\{\sqrt{\frac{1}{2}}\left(e_{k}+e_{l}\right):k<l,k,l=1,2,\cdots ,n\right\}\;$$

(13) $$\left\{\eta_{j}^{-}\right\}=\left\{\sqrt{\frac{1}{2}}\left(e_{k}-e_{l}\right):k<l,k,l=1,2,\cdots ,n\right\}\;$$

Another category of point-based filters, which choose points randomly, can be represented by PF. Although the performance is better, the heavy computational burden restricts their application in reality. For detailed rules of choosing points and the filtering process, refer to [34].

Being algebraically equivalent to the conventional filters, information filter has been widely used in multi-sensor information fusion. Instead of estimating the state $\hat{x}$ and the covariance P, it computes the information states $\hat{y}$ and information matrix Y at each time step. The relationships between $\hat{x},P$ and $\hat{y},Y$ are given by

(14) $$\hat{x}=Y^{-1}\hat{y}\;$$

(15) $$P=Y^{-1}\;$$

## 4. Sensor Selection

In this section, a sensor selection strategy is developed to address the problem 2 introduced in Section 2. Based on the classical control theory and the condition number [30], an observability metric is derived as the criterion to select the best combination of sensors. The continuous kind of the multi-sensor BOT system model (1) and (2) is given by

(16) $$\left\{ \begin{array}{l} {\dot{x}}_{p}=f_{p}(x_{p})={\left[\dot{x},\dot{y}\right]}^{T}\\ z=h(x_{p}) \end{array}\right.\;$$

where $x_{p}={\left[x,y\right]}^{T}$ denotes the position vector of the target and

$$h(x_{p})=\left[\begin{array}{c} h_{1}(x_{p})\\ h_{2}(x_{p}) \end{array}\right]=\left[\begin{array}{c} \beta_{1}\\ \beta_{2} \end{array}\right]=\left[\begin{array}{c} \arctan(\frac{x-s_{1}^{x}}{y-s_{1}^{y}})\\ \arctan(\frac{x-s_{2}^{x}}{y-s_{2}^{y}}) \end{array}\right]\;$$

The local nonlinear observability matrix of the multi-sensor BOT system can be computed as

(17) $$O=\left[\begin{array}{c} \nabla L_{f_{p}}^{0}(h_{1})\\ \nabla L_{f_{p}}^{1}(h_{1})\\ \vdots \\ \nabla L_{f_{p}}^{0}(h_{2})\\ \nabla L_{f_{p}}^{1}(h_{2})\\ \vdots \end{array}\right]=\left[\begin{array}{cc} \frac{y-s_{1}^{y}}{d_{1}^{2}} & \frac{-(x-s_{1}^{x})}{d_{1}^{2}}\\ O_{21}^{h_{1}} & O_{22}^{h_{1}}\\ \vdots & \vdots \\ \frac{y-s_{2}^{y}}{d_{2}^{2}} & \frac{-(x-s_{2}^{x})}{d_{2}^{2}}\\ O_{21}^{h_{2}} & O_{22}^{h_{2}}\\ \vdots & \vdots \end{array}\right]\;$$

where $\nabla L_{f_{p}}^{n}(h_{i})$ represents the n-order Lie derivative of $h_{i}$ along $f_{p}$ and

$$d_{i}=\sqrt{{\left(x-s_{i}^{x}\right)}^{2}+{\left(y-s_{i}^{y}\right)}^{2}},\;i=1,2\;$$

denotes the relative distance between the target and the *i*th sensor.

For simplicity, we neglect $O_{21}^{h_{1}}$, $O_{22}^{h_{1}}$, $O_{21}^{h_{2}}$, $O_{22}^{h_{2}}$, and other high-order terms, then the observability matrix can be simplified as

(18) $$O=\left[\begin{array}{cc} \frac{y-s_{1}^{y}}{d_{1}^{2}} & \frac{-(x-s_{1}^{x})}{d_{1}^{2}}\\ \frac{y-s_{2}^{y}}{d_{2}^{2}} & \frac{-(x-s_{2}^{x})}{d_{2}^{2}} \end{array}\right]\;$$

Through some basic trigonometric transform, Equation (18) can be rewritten as

(19) $$O=\left[\begin{array}{cc} \frac{\cos\beta_{1}}{d_{1}} & \frac{-\sin\beta_{1}}{d_{1}}\\ \frac{\cos\beta_{2}}{d_{2}} & \frac{-\sin\beta_{2}}{d_{2}} \end{array}\right]\;$$

The range information is weakly locally observable if the observability matrix O has full column rank. However, the rank could not tell us how well the tracking system is observable. In order to solve Problem 2, we use the inverse of the condition number of O, defined as the ratio of the smallest singular value to the largest one, given by

(20) $$OD=C^{-1}(O)=\frac{\sigma_{\min}(O)}{\sigma_{\max}(O)}\;$$

to describe the observability degree of the BOT system when $s_{1},s_{2}$ are used for tracking the target. A larger OD means a better observability and OD∈[0,1].

The singular values of O are equal to the square-root of the eigenvalues of the symmetric matrix, given as

(21) $$O=O^{T}O\;$$

and

(22) $$\{\begin{array}{c} \sigma_{\min}(O)=\sqrt{\lambda_{\min}(O)}\\ \sigma_{\max}(O)=\sqrt{\lambda_{\max}(O)} \end{array}\;$$

After some fundamental calculation, we can obtain the singular values of the observability matrix for the multi-sensor BOT system, as follows:

(23) $$\begin{array}{c} \sigma_{\min}(O)=\sqrt{\frac{(d_{1}^{2}+d_{2}^{2})-\sqrt{{\left(d_{1}^{2}+d_{2}^{2}\right)}^{2}-4d_{1}^{2}d_{2}^{2}\sin^{2}(\beta_{1}-\beta_{2})}}{2d_{1}^{2}d_{2}^{2}}}\\ \sigma_{\max}(O)=\sqrt{\frac{(d_{1}^{2}+d_{2}^{2})+\sqrt{{\left(d_{1}^{2}+d_{2}^{2}\right)}^{2}-4d_{1}^{2}d_{2}^{2}\sin^{2}(\beta_{1}-\beta_{2})}}{2d_{1}^{2}d_{2}^{2}}} \end{array}\;$$

By substituting Equation (23) into Equation (20), the observability metric, which is used to select the best pair of sensors, can be computed, as follows

(24) $$OD=\sqrt{\frac{(d_{1}^{2}+d_{2}^{2})-\sqrt{{\left(d_{1}^{2}+d_{2}^{2}\right)}^{2}-4d_{1}^{2}d_{2}^{2}\sin^{2}(\beta_{1}-\beta_{2})}}{(d_{1}^{2}+d_{2}^{2})+\sqrt{{\left(d_{1}^{2}+d_{2}^{2}\right)}^{2}-4d_{1}^{2}d_{2}^{2}\sin^{2}(\beta_{1}-\beta_{2})}}}\;$$

At time step *k*, the *OD* of each possible couple of sensors is calculated and the one with the largest value will be selected to track the target. Since the true target state is not usually available, we use the one-step prediction of the state, namely $x_{k|k-1}$, to compute *OD*.

**Remark** **2.** *It is obvious that* OD=0 *when* $\beta_{1}=\beta_{2}$ *or* $\beta_{1}=\beta_{2}+\pi$ *, which means that the selected pair of sensors should not at the same position or on a straight line with the target.*

**Remark** **3.** *This sensor selection strategy can help improve the observability of the tracking system as well as enhance the filtering precision. Besides, well-estimated state ensures that the process noise covariance estimation which is introduced in Section 5 could converge to the true value quickly.*

## 5. Adaptive Fifth-Degree Cubature Information Filter

The fifth-degree cubature information filter (FCIF) is algebraically equivalent to the fifth-degree CKF. In this section, two improvements, including the sensor selection strategy that was developed in Section 4 and a recursive process noise covariance estimation strategy are introduced into FCIF, thus both Problems 1 and 2 can be solved.

Assume that the posterior state and covariance estimates at time k−1 are ${\hat{x}}_{k-1|k-1}$ and $P_{k-1|k-1}$, the proposed AFCIF algorithm incorporates the following parts.

### 5.1. Time Update

The one-step prediction of the information state and covariance are given by

(25) $${\hat{y}}_{k|k-1}=Y_{k|k-1}{\hat{x}}_{k|k-1}=Y_{k|k-1}F{\hat{x}}_{k-1|k-1}\;$$

(26) $$Y_{k|k-1}=P_{k|k-1}^{-1}={\left(FP_{k-1|k-1}F^{T}+Q_{k-1}\right)}^{-1}\;$$

### 5.2. Measurement Update with Sensor Selection

In order to improve the observability of the range information and the precision of the target state estimation, the sensor selection strategy that was constructed in last section will be employed. By using this strategy, a pair of sensors that has the largest value of $C^{-1}(O)$ will be selected and the azimuth measurements that were received by them will be utilized to update the state and covariance.

Assume that the sensor network has $N_{k}$ individual sensors at time *k* and there will be $\frac{N_{k}(N_{k}-1)}{2}$ kinds of combinations, including $\left(s_{1},s_{2}),(s_{1},s_{3}),\cdots ,(s_{1},s_{N_{k}}),\cdots ,(s_{N_{k}-1},s_{N_{k}}\right)$, then we calculate $C^{-1}(O)$ of all combinations and choose the one that achieves the largest value.

In the measurement update, the generated cubature points and propagated ones are given by

(27) $$\chi_{i,k|k-1}=\sqrt{P_{k|k-1}}\xi_{i}+{\hat{x}}_{k|k-1}\;$$

(28) $$z_{i,k|k-1}=h_{k}(\chi_{i,k|k-1})\;$$

the predicted measurement is

(29) $${\hat{z}}_{k|k-1}=\sum_{i=1}^{2n^{2}+1}\omega_{i}z_{i,k|k-1}\;$$

According to [19], once the measurement at time *k* is available, the filtered information state and the covariance can be computed as

(30) $${\hat{y}}_{k|k}={\hat{y}}_{k|k-1}+Y_{k|k-1}P_{k|k-1}^{xz}R^{-1}\left[z_{k}-h_{k}({\hat{x}}_{k|k-1})+P_{k|k-1}^{xz,T}Y_{k|k-1}^{T}{\hat{x}}_{k|k-1}\right]\;$$

(31) $$Y_{k|k}=Y_{k|k-1}+Y_{k|k-1}P_{k|k-1}^{xz}R^{-1}P_{k|k-1}^{xz,T}Y_{k|k-1}^{T}\;$$

where the cross covariance matrix is given by

(32) $$P_{k|k-1}^{xz}=\sum_{i=1}^{2n^{2}+1}\omega_{i}(\chi_{i,k|k-1}-{\hat{x}}_{k|k-1}){\left(z_{i,k|k-1}-{\hat{z}}_{k|k-1}\right)}^{T}\;$$

At last the state and covariance can be recovered by

(33) $${\hat{x}}_{k|k}=Y_{k|k}^{-1}{\hat{y}}_{k|k}\;$$

(34) $$P_{k|k}=Y_{k|k}^{-1}\;$$

### 5.3. Recursive Estimation of Process Noise Covariance

In reality, statistic of the process noise is not usually available as described in Problem 1, so we build a recursive estimation strategy through covariance matching principle.

Consider one-step prediction of state ${\hat{x}}_{k|k-1}$ at time k−1 and the filtered state ${\hat{x}}_{k|k}$ at time *k*, the residual between them can be represented by

(35) $$\zeta_{k}={\hat{x}}_{k|k}-{\hat{x}}_{k|k-1}\;$$

Given the residual data from time k−N+1 to time *k*, the mean and the covariance of $\zeta_{k}$ can be estimated by

(36) $${\bar{\zeta }}_{k}=\frac{1}{N}\sum_{i=k-N+1}^{k}\zeta_{i}\;$$

(37) $$\Sigma_{\zeta_{k}}=\frac{1}{N-1}\sum_{i=k-N+1}^{k}\left(\zeta_{i}-{\bar{\zeta }}_{k}\right){\left(\zeta_{i}-{\bar{\zeta }}_{k}\right)}^{T}\;$$

where *N* is an adjustable parameter. Inspired by the measurement noise covariance update rule derived in [24], we can obtain the following results.

**Theorem** **1.** *Under the premise that the covariance of process noise stay constant, the recursive relation between* $Q_{k-1}$ *and* $Q_{k}$ *can be described as*

(38) $$Q_{k}=\frac{N-1}{N}Q_{k-1}+\Delta Q_{k}\;$$

*where*

(39) $$\Delta Q_{k}=\frac{1}{N-1}\left(\zeta_{k}-{\bar{\zeta }}_{k}\right){\left(\zeta_{k}-{\bar{\zeta }}_{k}\right)}^{T}-\frac{1}{N}\left(FP_{k-1|k-1}F^{T}-P_{k|k}\right)\;$$

**Proof.**  Under the condition that the process noise covariance is constant, the expectation of Equation (37) is given by [22]

(40) $$E(\Sigma_{\zeta })=\frac{1}{N}\sum_{i=k-N+1}^{k}\left(FP_{i-1|i-1}F^{T}-P_{i|i}\right)+Q_{k}\;$$

Combine Equations (37) and (40), the covariance of process noise can be approximately calculated by

(41) $$Q_{k}=\frac{1}{N-1}\sum_{i=k-N+1}^{k}(\zeta_{i}-{\bar{\zeta }}_{k}){\left(\zeta_{i}-{\bar{\zeta }}_{k}\right)}^{T}-\frac{1}{N}\sum_{i=k-N+1}^{k}\left(FP_{i-1|i-1}F^{T}-P_{i|i}\right)\;$$

Similarly, $Q_{k-1}$ can be computed as

(42) $$Q_{k-1}=\frac{1}{N-2}\sum_{i=k-N+1}^{k-1}(\zeta_{i}-{\bar{\zeta }}_{k}){\left(\zeta_{i}-{\bar{\zeta }}_{k}\right)}^{T}-\frac{1}{N-1}\sum_{i=k-N+1}^{k-1}\left(FP_{i-1|i-1}F^{T}-P_{i|i}\right)\;$$

Through some simple mathematical transformation, Equation (41) can be rewritten as the following form

(43) $$Q_{k}=\frac{N-1}{N}X_{k}+\Delta Q_{k}\;$$

where

(44) $$X_{k}=\frac{N}{{\left(N-1\right)}^{2}}\sum_{i=k-N+1}^{k-1}(\zeta_{i}-{\bar{\zeta }}_{k}){\left(\zeta_{i}-{\bar{\zeta }}_{k}\right)}^{T}-\frac{1}{N-1}\sum_{i=k-N+1}^{k-1}\left(FP_{i-1|i-1}F^{T}-P_{i|i}\right)\;$$

If *N* is large enough, the difference between $N/{\left(N-1\right)}^{2}$ and 1/(N−2) is negligible. So, $X_{k}$ can be approximated as

(45) $$X_{k}\approx \frac{1}{N-2}\sum_{i=k-N+1}^{k-1}(\zeta_{i}-{\bar{\zeta }}_{k}){\left(\zeta_{i}-{\bar{\zeta }}_{k}\right)}^{T}-\frac{1}{N-1}\sum_{i=k-N+1}^{k-1}\left(FP_{i-1|i-1}F^{T}-P_{i|i}\right)\;$$

Obviously, the above equation has the equivalent form with $Q_{k-1}$, therefore we can obtain Equation (38). This completes the proof. Similarly, the recursive estimation equation of ${\bar{\zeta }}_{k}$ is given by

(46) $${\bar{\zeta }}_{k}=\frac{N-1}{N}{\bar{\zeta }}_{k-1}+\frac{1}{N}\zeta_{k}\;$$

So, we can use Equations (35), (38), (39) and (46) to update the covariance of process noise. □

**Remark** **4.** *The parameter N in Equations (38) and (46) needs to be adjusted when this noise covariance estimation strategy is used in reality. A large N means that relatively accurate statistical information of the process noise can be obtained and the updated* $Q_{k}$ *is very close to* $Q_{k-1}$ *. A small N, however, indicates that there is no knowledge of the process noise available and* $Q_{k}$ *is more dependent on the newly estimated state.*

### 5.4. Adaptive Fifth-Degree Cubature Information Filter for Multi-Sensor Estimation

When compared with the conventional Bayesian filters, the information-form ones are preferred in multi-sensor tracking due to the simpler measurement update process. In the information filter, the measurements received by the sensors at different locations can be fused by adding each measurement contribution to the information state and matrix, and then the filtered information state and matrix can be calculated as

(47) $${\hat{y}}_{k|k}={\hat{y}}_{k|k-1}+\sum_{i=1}^{2}P_{k|k-1}^{-1}P_{i,k|k-1}^{xz}R_{i}^{-1}\left[z_{k,i}-h_{k,i}({\hat{x}}_{k|k-1})+P_{i,k|k-1}^{xz,T}P_{k|k-1}^{-T}{\hat{x}}_{k|k-1}\right]\;$$

(48) $$Y_{k|k}=Y_{k|k-1}+\sum_{i=1}^{2}P_{k|k-1}^{-1}P_{i,k|k-1}^{xz}R_{i}^{-1}P_{i,k|k-1}^{xz,T}P_{k|k-1}^{-T}\;$$

where $P_{i,k|k-1}^{xz}$ is the *i*th column of $P_{k|k-1}^{xz}$, $R_{i}$ is the measurement noise covariance of the *i*th sensor and $z_{k,i}$ is the measurement of the *i*th sensor at time *k*.

## 6. Convergence Analysis

Now, we analyze the convergence of the novel algorithm when used in multi-sensor BOT for state estimation. Firstly, we define the posterior state estimation error, the predicted state error and measurement error at time *k*, respectively, by

(49) $${\tilde{x}}_{k}=x_{k}-{\hat{x}}_{k|k}\;$$

(50) $${\tilde{x}}_{k|k-1}=x_{k}-{\hat{x}}_{k|k-1}\;$$

(51) $${\tilde{z}}_{k}=z_{k}-h_{k}({\hat{x}}_{k|k-1})\;$$

and the relationships between them are described, as follows

(52) $${\tilde{x}}_{k}={\tilde{x}}_{k|k-1}-K_{k}{\tilde{z}}_{k}\;$$

(53) $${\tilde{x}}_{k|k-1}=F{\tilde{x}}_{k-1}\;$$

For simplicity, Equation (51) can be transformed into the following linearized form

(54) $${\tilde{z}}_{k}=\alpha_{k}H_{k}{\hat{x}}_{k|k-1}\;$$

where $\alpha_{k}$ is a diagonal matrix. Since the CKF is a kind of derivative-free algorithm and no linearization error can be produced, we use this exact form just for the convenience of theory analysis. Here, instead of $H_{k}$, $\alpha_{k}H_{k}$ becomes the measurement matrix.

**Theorem** **2.** *The proposed AFCIF algorithm is stable and can guarantee the estimation of the multi-sensor BOT system convergent if the following conditions hold*

- The filtered covariance matrix is bounded.
  (55) $$aI_{n}\leq P_{k-1|k-1}^{-1}\leq bI_{n}\;$$
- The state transition matrix is invertible and bounded.
  (56) ‖F‖≤c 
- The updated process noise covariance satisfies
  (57) $$(1-\lambda )\underline{\sigma }(FP_{k-1|k-1}F^{T}+Q_{k-1})\geq \bar{\sigma }{\left(F\right)}^{2}\bar{\sigma }(P_{k-1|k-1})\;$$
  where a,b,c are positive real numbers, 0<λ<1, $\underline{\sigma }$, and $\bar{\sigma }$ denote the minimum and maximum singular values.

**Proof.**  The Lyapunov function at time *k* is defined as

(58) $$V({\tilde{x}}_{k})={\tilde{x}}_{k}^{T}P_{k|k}^{-1}{\tilde{x}}_{k}\;$$

Similarly,

(59) $$V({\tilde{x}}_{k-1})={\tilde{x}}_{k-1}^{T}P_{k-1|k-1}^{-1}{\tilde{x}}_{k-1}\;$$

and it is obvious that V(0)=0.

The Kalman gain and the inverse of the error covariance can be calculated as

(60) $$K_{k}=P_{k|k-1}H_{k}^{T}\alpha_{k}{\left(\alpha_{k}H_{k}P_{k|k-1}H_{k}^{T}\alpha_{k}+R\right)}^{-1}=P_{k|k}H_{k}^{T}\alpha_{k}R^{-1}\;$$

(61) $$P_{k|k}^{-1}=P_{k|k-1}^{-1}+H_{k}^{T}\alpha_{k}R^{-1}\alpha_{k}H_{k}\;$$

$V({\tilde{x}}_{k})$ becomes

(62) $$V({\tilde{x}}_{k})={\tilde{x}}_{k|k-1}^{T}P_{k|k}^{-1}{\tilde{x}}_{k|k-1}-{\tilde{x}}_{k|k-1}^{T}H_{k}^{T}\alpha_{k}R^{-1}{\tilde{z}}_{k}-{\tilde{z}}_{k}^{T}R^{-1}\alpha_{k}H_{k}{\tilde{x}}_{k|k-1}+{\tilde{z}}_{k}^{T}R^{-1}\alpha_{k}H_{k}P_{k|k}H_{k}^{T}\alpha_{k}R^{-1}{\tilde{z}}_{k}\;$$

by substituting (60) into (52) and (52) into (58). Then, substitute (61) into (62), we obtain

(63) $$\begin{array}{ll} V({\tilde{x}}_{k})= & {\tilde{x}}_{k|k-1}^{T}P_{k|k-1}^{-1}{\tilde{x}}_{k|k-1}+{\tilde{x}}_{k|k-1}^{T}H_{k}^{T}\alpha_{k}R^{-1}\alpha_{k}H_{k}{\tilde{x}}_{k|k-1}\\ & -{\tilde{x}}_{k|k-1}^{T}H_{k}^{T}\alpha_{k}R^{-1}{\tilde{z}}_{k}-{\tilde{z}}_{k}^{T}R^{-1}\alpha_{k}H_{k}{\tilde{x}}_{k|k-1}+{\tilde{z}}_{k}^{T}R^{-1}\alpha_{k}H_{k}P_{k|k}H_{k}^{T}\alpha_{k}R^{-1}{\tilde{z}}_{k} \end{array}$$

Combined with (26), (53), and (54), (63) becomes

(64) $$\begin{array}{ll} V({\tilde{x}}_{k}) & ={\tilde{x}}_{k-1}^{T}F^{T}{\left(FP_{k-1|k-1}F^{T}+Q_{k-1}\right)}^{-1}F{\tilde{x}}_{k-1}-{\tilde{z}}_{k}^{T}(R^{-1}-R^{-1}\alpha_{k}H_{k}P_{k|k}H_{k}^{T}\alpha_{k}R^{-1}){\tilde{z}}_{k}\\ & ={\tilde{x}}_{k-1}^{T}F^{T}{\left(FP_{k-1|k-1}F^{T}+Q_{k-1}\right)}^{-1}F{\tilde{x}}_{k-1}-{\tilde{z}}_{k}^{T}P_{zz}^{-1}{\tilde{z}}_{k}\\ & \leq {\tilde{x}}_{k-1}^{T}F^{T}{\left(FP_{k-1|k-1}F^{T}+Q_{k-1}\right)}^{-1}F{\tilde{x}}_{k-1} \end{array}$$

where $R^{-1}-R^{-1}\alpha_{k}H_{k}P_{k|k}H_{k}^{T}\alpha_{k}R^{-1}=P_{zz}^{-1}$, as shown in [12]. Then according to (57), (59), and (64),

(65) $$V({\tilde{x}}_{k})-(1-\lambda )V({\tilde{x}}_{k-1})\leq 0\;$$

further

(66) $$0\leq a{\tilde{x}}_{k}^{T}{\tilde{x}}_{k}\leq V({\tilde{x}}_{k})\leq {\left(1-\lambda \right)}^{k}V({\tilde{x}}_{0})\;$$

Since

(67) $$0\leq a\underset{k\to \infty }{\lim}({\tilde{x}}_{k}^{T}{\tilde{x}}_{k})\leq \underset{k\to \infty }{\lim}V({\tilde{x}}_{k})\leq V({\tilde{x}}_{0})\underset{k\to \infty }{\lim}{\left(1-\lambda \right)}^{k}=0\;$$

we can obtain

(68) $$\underset{k\to \infty }{\lim}({\hat{x}}_{k}-x_{k})=0\;$$

Therefore, the state estimation error will converge to 0 over time. This completes the proof. □

**Remark** **5.** *The sensor selection strategy helps make the tracking system locally observable, which ensures that Condition I is satisfied. Condition II is naturally satisfied according to Equation (3).*

**Remark** **6.** *An easy way to keep the filtering stability is to use a sufficiently large* $Q_{k-1}$ *. However, this may cause degenerated estimation performance. On the contrary, a small* $Q_{k-1}$ *could lead to unstable performance, even though it may produce better filtering precision at some time. The proposed AFCIF algorithm can provide accurate estimation of* $Q_{k}$ *as* k→∞ *, thus both the precision and stability can be guaranteed.*

## 7. Simulation Analysis

In this section, the proposed AFCIF algorithm is compared with FCIF through 500 Monte Carlo runs in a multi-sensor BOT scenario. The motion of the target follows the dynamic equation that is described by (1). The sample interval ΔT=1 s and the target’s motion lasts for 150 s. The initial target state is $x_{0}={\left[-10\;\mathrm{km},\;10\;\mathrm{km},\;50\;m/s,\;-100\;m/s\right]}^{T}$ and the initial covariance is set to be $P_{0}=\mathrm{diag}([10^{4}\;m^{2},\;10^{4}\;m^{2},\;10^{2}\;m^{2}/s^{2},\;10^{2}\;m^{2}/s^{2}])$. The initial state estimate ${\hat{x}}_{0}$ follows the Gaussian distribution $N({\hat{x}}_{0};\;x_{0},\;P_{0})$. We assume that a group of four passive sensors are ready to track the target using bearing measurements and only two of them will be selected at each time. The fixed localizations of the four sensors are given by

$$\begin{array}{l} s_{1}^{x}=4\;\mathrm{km},\;s_{1}^{y}=-10\;\mathrm{km}\;s_{2}^{x}=5\;\mathrm{km},\;s_{2}^{y}=-7\;\mathrm{km}\\ s_{3}^{x}=0\;\mathrm{km},\;s_{3}^{y}=-10\;\mathrm{km}\;s_{4}^{x}=-8\;\mathrm{km},\;s_{4}^{y}=-7\;\mathrm{km} \end{array}$$

The measurement noises of all the sensors are assumed to be white Gaussian with covariance $R_{i}={\left(0.1^{\circ }\right)}^{2},i=1,2,3,4$. The process noise is also assumed to be zero-mean Gaussian with unknown covariance $Q_{\mathrm{true}}=\mathrm{diag}([100\;m^{2},\;100\;m^{2},\;1\;m^{2}\cdot s^{-2},\;1\;m^{2}\cdot s^{-2}])$. The prior process noise covariance is set to be $Q_{\mathrm{big}}=100Q_{\mathrm{true}}$ and $Q_{\mathrm{small}}=0.01Q_{\mathrm{true}}$. Under these two initializations, we conduct simulations using the standard FCIF and the proposed AFCIF with different combinations of sensors.

We use the root-mean-square error (RMSE) as our performance metric to compare the accuracy and stability of these two algorithms. The RMSE of position at time *k* is defined as

(69) $$\mathrm{RMS}E_{k}^{\mathrm{pos}}=\sqrt{\frac{1}{M}\sum_{i=1}^{M}{\left({\hat{x}}_{k}^{i}-x_{k}^{i}\right)}^{2}+{\left({\hat{y}}_{k}^{i}-y_{k}^{i}\right)}^{2}}\;$$

where *M* is the overall number of Monte Carlo runs, $\left(x_{k}^{i},y_{k}^{i}\right)$ and $\left({\hat{x}}_{k}^{i},{\hat{y}}_{k}^{i}\right)$ are the true position and estimated position of the target, respectively, in the *i*th run at time *k*. Similarly, the RMSE of velocity at time *k* is defined as

(70) $$\mathrm{RMS}E_{k}^{\mathrm{vel}}=\sqrt{\frac{1}{M}\sum_{i=1}^{M}{\left({\hat{\dot{x}}}_{k}^{i}-{\dot{x}}_{k}^{i}\right)}^{2}+{\left({\hat{\dot{y}}}_{k}^{i}-{\dot{y}}_{k}^{i}\right)}^{2}}\;$$

where $\left({\dot{x}}_{k}^{i},{\dot{y}}_{k}^{i}\right)$ and $\left({\hat{\dot{x}}}_{k}^{i},{\hat{\dot{y}}}_{k}^{i}\right)$ are true velocity and estimated velocity of the target, respectively, in the *i*th run at time *k*.

The RMSE of process noise covariance is defined as

(71) $$\mathrm{RMS}E_{k}^{\mathrm{cov}}=\sqrt{\frac{1}{M}\sum_{i=1}^{M}\sum_{j=1}^{4}{\left({\hat{Q}}_{k,i}^{jj}-Q_{true}^{jj}\right)}^{2}}\;$$

where $Q_{\mathrm{true}}^{jj}$ and ${\hat{Q}}_{k,i}^{jj}$ represent the *j*th component of the true process noise covariance and the estimated one in the *i*th run at time *k*. Here, without considering units, we define this $\mathrm{RMS}E_{k}^{\mathrm{cov}}$ by integrating each diagonal element in the covariance matrix.

In the target tracking literature, the posterior Cramér-Rao lower bound (PCRLB) has been extensively used as an indication of the best possible performance that a filtering algorithm can get close to, but typically not achieve. The PCRLB for the RMSE of position and velocity at time *k* are computed, as follows:

(72) $$\mathrm{PCRLB}(\mathrm{RMS}E_{k}^{\mathrm{pos}})=\sqrt{J_{k}^{-1}(1,1)+J_{k}^{-1}(2,2)}\;$$

(73) $$\mathrm{PCRLB}(\mathrm{RMS}E_{k}^{\mathrm{vel}})=\sqrt{J_{k}^{-1}(3,3)+J_{k}^{-1}(4,4)}\;$$

where $J_{k}^{-1}(i,i)$ denotes the *i*th element of the principal diagonal of $J_{k}^{-1}$. In [35], Tichavský developed the recursive formula below for calculating the Fisher information matrix, namely $J_{k}$, at each time step:

(74) $$J_{k}=D_{k}^{22}-D_{k}^{21}{\left[J_{k-1}+D_{k}^{11}\right]}^{-1}D_{k}^{12}\;$$

where

$$\begin{array}{l} D_{k}^{11}=E\left\{F_{k}^{T}Q_{k}^{-1}F_{k}\right\}=F^{T}Q_{\mathrm{true}}^{-1}F\\ D_{k}^{12}=-E\left\{F_{k}^{T}Q_{k}^{-1}\right\}=-F^{T}Q_{\mathrm{true}}^{-1}\\ D_{k}^{21}=-E\left\{Q_{k}^{-1}F_{k}\right\}=-Q_{\mathrm{true}}^{-1}F\\ D_{k}^{22}=E\left\{H_{k}^{T}R_{k}^{-1}H_{k}\right\}+Q_{k}^{-1}=H_{k}^{T}R^{-1}H_{k}+Q_{\mathrm{true}}^{-1} \end{array}$$

and $H_{k}$ is the Jacobian of the measurement function.

**Figure 2:**
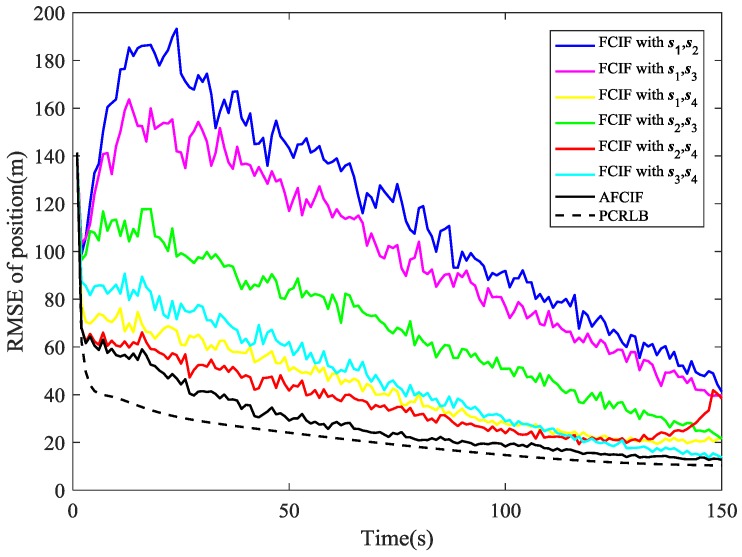
Comparison of RMSE (position estimation) when the initial process noise covariance is set to be larger than the true value.

**Figure 3:**
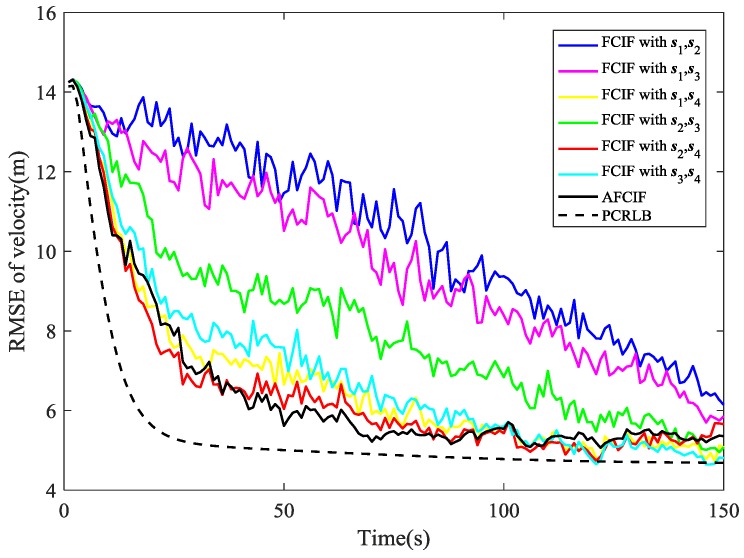
Comparison of RMSE (velocity estimation) when the initial process noise covariance is set to be larger than the true value.

**Figure 4:**
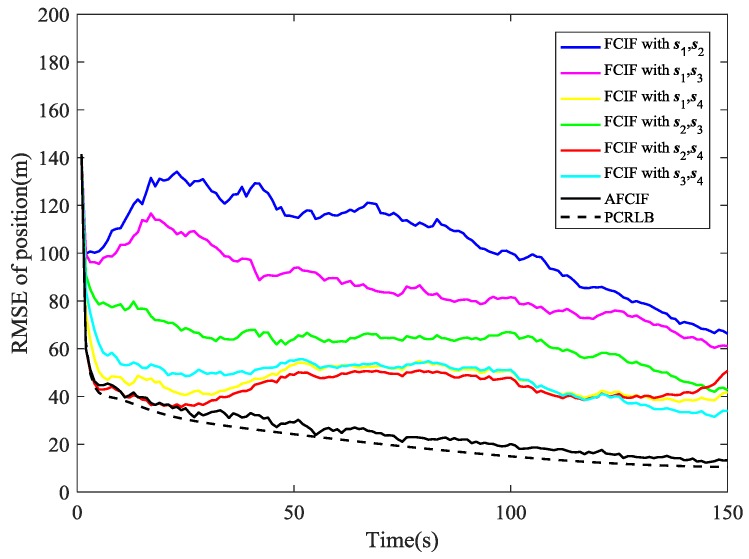
Comparison of RMSE (position estimation) when the initial process noise covariance is set to be smaller than the true value.

**Figure 5:**
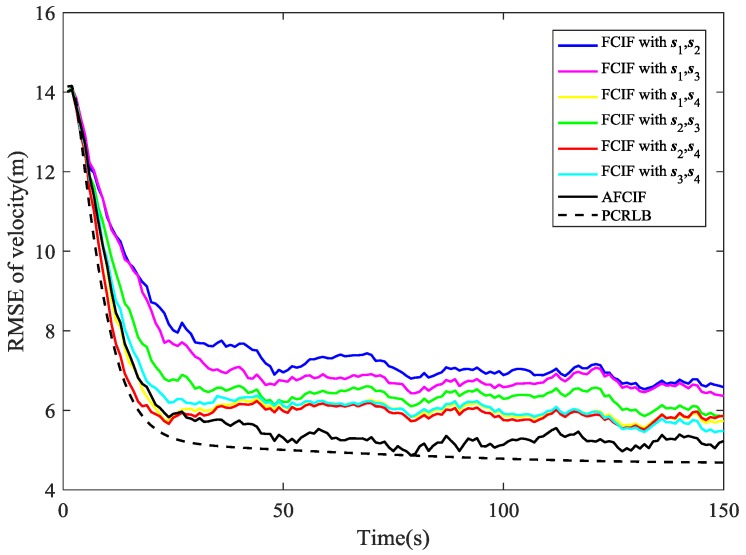
Comparison of RMSE (velocity estimation) when the initial process noise covariance is set to be smaller than the true value.

**Figure 6:**
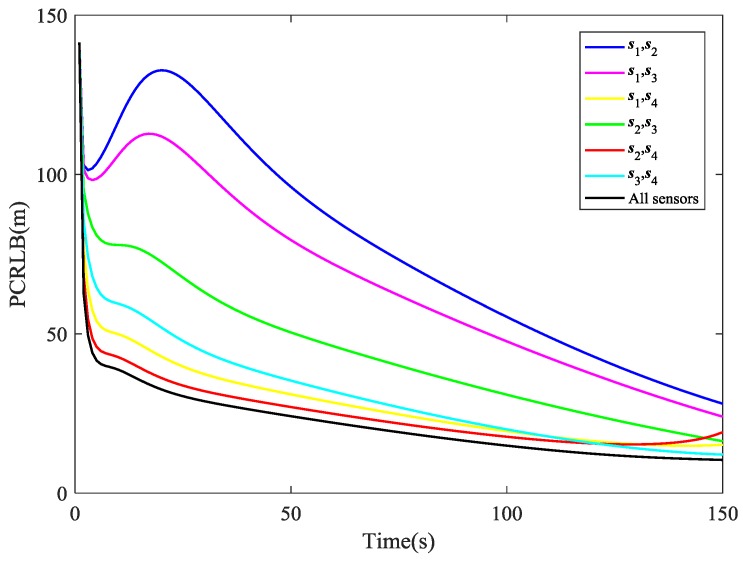
Comparison of posterior Cramér-Rao lower bound (PCRLB).

**Figure 7:**
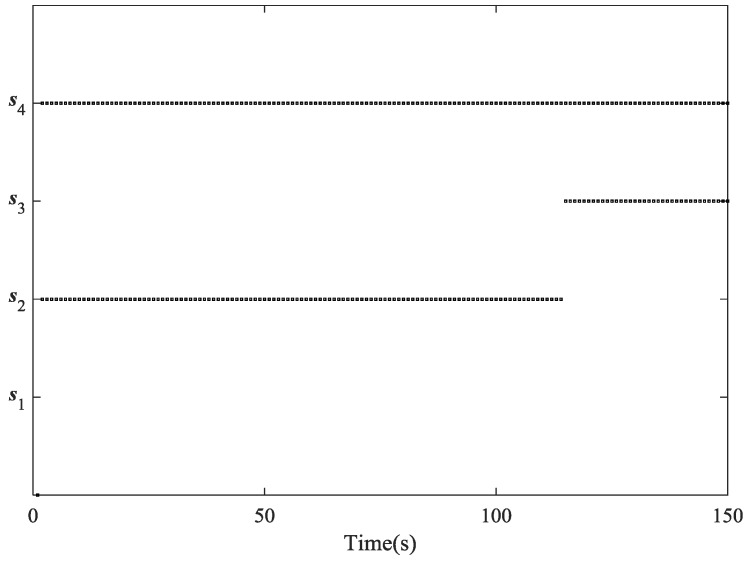
Sensor selection at each time step.

**Figure 8:**
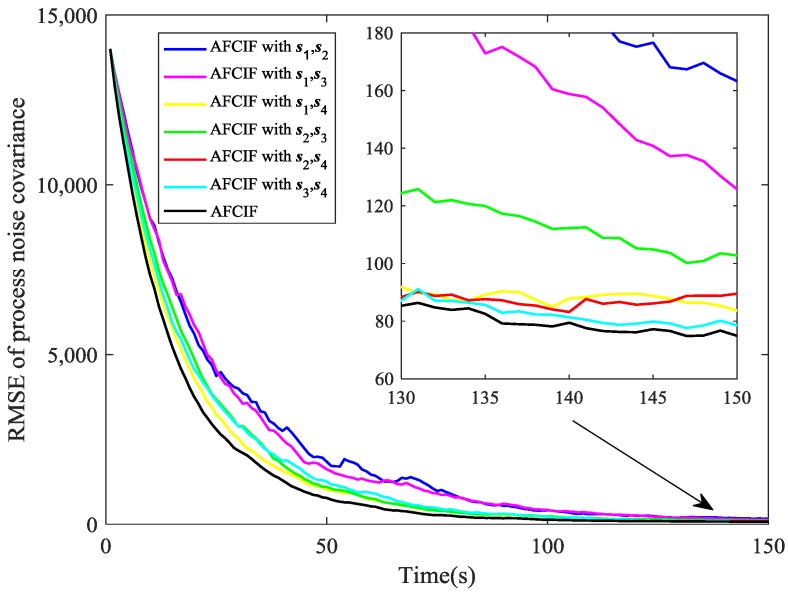
Comparison of RMSE (noise estimation) when the initial process noise covariance is set to be larger than the true value.

**Figure 9:**
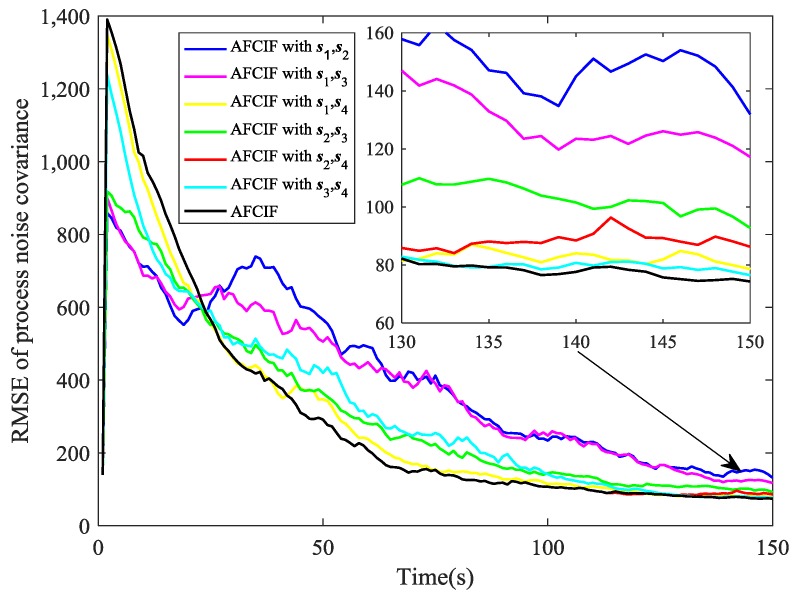
Comparison of RMSE (noise estimation) when the initial process noise covariance is set to be smaller than the true value.

The simulation results are illustrated in Figure 2, Figure 3, Figure 4, Figure 5, Figure 6, Figure 7, Figure 8 and Figure 9. The PCRLB curves in Figure 2, Figure 4 and Figure 3, Figure 5 denote the posterior Cramér-Rao lower bounds for the RMSEs of position and velocity, respectively. The PCRLB curves shown in the figures are based on the situation when all the sensors are selected for tracking the target. Hence, the Jacobian of the measurement function used for computing the PCRLBs is given by

(75) $$H_{k}=\left[\begin{array}{cccc} \frac{y_{k}-s_{1}^{y}}{d_{k,1}^{2}} & \frac{-(x_{k}-s_{1}^{x})}{d_{k,1}^{2}} & 0 & 0\\ \frac{y_{k}-s_{2}^{y}}{d_{k,2}^{2}} & \frac{-(x_{k}-s_{2}^{x})}{d_{k,2}^{2}} & 0 & 0\\ \frac{y_{k}-s_{3}^{y}}{d_{k,3}^{2}} & \frac{-(x_{k}-s_{3}^{x})}{d_{k,3}^{2}} & 0 & 0\\ \frac{y_{k}-s_{4}^{y}}{d_{k,4}^{2}} & \frac{-(x_{k}-s_{4}^{x})}{d_{k,4}^{2}} & 0 & 0 \end{array}\right]\;$$

where $d_{k,i}=\sqrt{{\left(x_{k}-s_{i}^{x}\right)}^{2}+{\left(y_{k}-s_{i}^{y}\right)}^{2}},\;i=1,2,3,4$ denotes the relative distance between the *i*th sensor and the target at time *k*.

As depicted in Figure 2, the estimation performance of AFCIF on target position is better than that of each standard FCIF when the initial estimate of process noise covariance is much larger. Although experiencing some obvious fluctuations, the RMSE curves of the FCIFs with different combinations of sensors stay descending until the last second, except the one with $s_{1},\;s_{4}$ and the one with $s_{2},\;s_{4}$ become gradually divergent at the last 20 s. However, the curve of the AFCIF keeps the downtrend with smaller fluctuations, which indicates better filtering stability. As for the estimation performance on target velocity, it can be seen in Figure 3 that the RMSE curves of AFCIF and FCIFs with $s_{1},s_{4}$, $s_{2},s_{4}$, and $s_{3},s_{4}$ almost overlap in the last 50 s. Overall, when the initial process noise covariance is larger than the true value, the performance difference between FCIF and AFCIF is decreasing over time.

While if the initial value of process noise covariance is smaller than the true one, the simulation results are different. As shown in Figure 4 and Figure 5, the RMSE curves of AFCIF are easy to be distinguished from those of the FCIFs and the performance differences keep a growing tendency in most of the time. This can be explained by the convergence condition that is shown in (57). A smaller $Q_{k-1}$ will lead to unstable and divergent results. With no accurate statistic of the process noise, we can use a larger initial value to guarantee the stability and convergence of the filtering algorithm. No matter how the initial process noise covariance is assumed, the RMSE curves of AFCIF approach the PCRLB gradually over time (as shown in Figure 2, Figure 3, Figure 4 and Figure 5), which validates the superiority of AFCIF and the correctness of the theoretical analysis.

Under the condition of unknown process noise covariance, the performance gap between FCIF and AFCIF is due to the model mismatching, which means that the standard FCIF always uses an inaccurate process noise covariance in the filtering procedure. However, the proposed AFCIF estimates and modifies the covariance online, which can guarantee the estimation results stable and convergent. In our simulation, since the initial estimate of the covariance (no matter $Q_{\mathrm{big}}$ or $Q_{\mathrm{small}}$) has large deviation from the true value, the parameter *N* should be small. Otherwise the estimation will reach convergence more slowly. All of the simulation results are based on N=10.

Now we compare the tracking performance while using different combinations of sensors. From the comparison of PCRLB for RMSE of position shown in Figure 6, it is easy to notice that $s_{2},\;s_{4}$ is the best pair of sensors in most of the time and $s_{1},\;s_{2}$ is the last choice. From about the 120th second to the end of the simulation, however, the performance of tracking the target with $s_{2},\;s_{4}$ begins to degrade and $s_{3},\;s_{4}$ turns to be the best combination. These results are corresponding to the illustration in Figure 7, which shows the specific sensor selection of the AFCIF at each time step. Another valuable information in Figure 6 is that the tracking performance using $s_{1},\;s_{4}$, $s_{2},\;s_{4}$ and $s_{3},\;s_{4}$ are very close to that using all the sensors. The strategy of sensor selection helps reach the best tracking performance with as few sensors as possible, thus the sensor resources can be utilized reasonably and sufficiently.

In Figure 8 and Figure 9, we compare the performance on estimating process noise covariance of AFCIF with sensor selection and AFCIFs with fixed combinations of sensors. Once again, sensor selection strategy is proved to make the estimation of target state more accurate and this also facilitates the estimation of the process noise covariance.

Table 1 shows the computation time of the algorithms relative to that of FCIF. AFCIF runs a litter slower, since it incorporates the procedures of selecting the best pair of sensors and estimating the process noise covariance. While considering the prominent improvement over FCIF, this added computation burden of AFCIF is ignorable.

## 8. Conclusions

In this paper, we have proposed an adaptive fifth-degree cubature information filter (AFCIF) for multi-sensor BOT. Our main contributions are summarized, as follows:

(1) A sensor selection strategy based on the observability theory is developed for multi-sensor BOT to choose the optimal combination of sensors to track the target at each sample interval. With the given sensors at different locations, this strategy can help the tracking system reach the best possible observability, which will enhance the filtering stability and accuracy. By using this strategy, the sensor resource can also be made the best use of.
(2) A novel nonlinear estimation algorithm is constructed within the information filtering framework. Using the newly introduced fifth-degree cubature rule, this algorithm can approximate the multidimensional integrals existing in Bayesian filtering with considerable accuracy.
(3) In order to deal with the state estimation of nonlinear system that is corrupted by Gaussian noise with unknown covariance, a recursive covariance estimation procedure derived through the covariance matching principle is embedded into the proposed algorithm. Thus, the statistical property of process noise, which is not usually available in reality, can be estimated and modified in time. As a result, the estimated covariance will get close to the true value over time, which can improve the estimation performance of the algorithm.

The efficacy of the proposed algorithm is verified by the simulation results in a multi-sensor BOT scenario. Under the circumstance of unknown process noise covariance, the target state can still be well estimated. Through the sensor selection strategy, the best combination of sensors with the lowest PCRLB and the highest degree of observability can always be selected, which could further enhance the estimation performance on target state and process noise covariance.

## Figures and Tables

**Table 1:** Relative Computation Times of the algorithms.

Algorithm	Relative Computation Time
FCIF	1
AFCIF	1.7
